# Subcutaneous glucagon infusion and continuous glucose monitoring enable effective management of hypoglycemia in a patient with IGF-2-producing hemangiopericytoma

**DOI:** 10.1186/s40842-017-0053-0

**Published:** 2018-01-09

**Authors:** Eric D. Buras, Emily Weatherup, Jennifer Wyckoff

**Affiliations:** Department of Internal Medicine; Division of Metabolism, Endocrinology and Diabetes, University of Michigan, Domino’s Farms Lobby C. 24 Frank Lloyd Wright Drive, Ann Arbor, MI 48106-9484 USA

**Keywords:** Insulin like growth factor (IGF)-2, Hemangiopericytoma, Glucagon, Continuous glucose monitoring

## Abstract

**Background:**

Ectopic insulin-like growth factor (IGF)-2 production is a rare complication of an array of epithelial and mesenchymal tumors, and can clinically manifest as life-threatening hypoglycemia.

**Case presentation:**

A 49-year-old woman with 13-year history of metastatic hemangiopericytoma, previously treated with multiple rounds of chemotherapy and palliative radiation, presented to the emergency department after a hypoglycemic seizure. On arrival, glucose was 18 mg/dL (1.0 mmol/L) and required continuous dextrose infusion for maintenance within normal limits. Insulin was <2.0 μU/mL, C-peptide 0.1 ng/mL, and beta-hydroxybutyrate <0.2 mmol/L. Random cortisol was 21 μg/dL; sulfonylurea screen, and insulin antibodies were negative. IGF-2 level was 1320 ng/mL; IGF-1 was within normal limits and IGF binding protein (BP)-3 suppressed. Dexamethasone, started at 6 mg twice daily, allowed discontinuation of the glucose infusion. Given concern for nocturnal hypoglycemia, and patient interest in steroid-sparing anti-hypoglycemic regimen, she was also started on overnight continuous subcutaneous glucagon infusion via insulin pump. She was discharged with instructions to maintain a diet high in complex carbohydrates during the day, while utilizing glucagon pump at night. She was also started on continuous glucose monitoring system (CGMS) with an alarm to warn of hypoglycemia. Glucagon infusion rate was later titrated based on CGMS readings. Abdominal CT revealed increasing size of a right upper quadrant mass not previously subjected to radiotherapy. After radiation to this area, hypoglycemia improved, allowing further glucagon titration. In parallel, IGF-2 level declined to 380 ng/mL.

**Conclusions:**

Ectopic IGF-2 production is a rare but often fatal complication of many cancers, and should be considered on the differential diagnosis in patients with malignancy and unexplained hypoglycemia. Once hypoglycemia is diagnosed, patients often have end-stage disease. While treatment of the causative tumor is the only definitive intervention, anti-hypoglycemia therapy is a life-saving, temporizing measure. In this case, the patient attained euglycemia and survived 3 months after presentation before ultimately succumbing to other malignancy-related complications. Given efficacy in management of hypoglycemia while awaiting definitive tumor-directed therapy, we submit nighttime subcutaneous glucagon infusion and CGMS are valuable additions to the physician’s armamentarium in managing this condition.

## Background

In a small minority of patients, malignancies including hepatocellular carcinoma, solitary fibrous tumors and mesothelioma, produce IGF-2 and release it into circulation [1, 2]. Dynkevich et al. coined the term IGF-2oma to describe these tumors [3]. Due to cross-reactivity at the insulin receptor, ectopic IGF-2 can precipitate a clinical syndrome that mimics the fasting hypoglycemia characteristic of patients with insulinoma [4, 5]. In rare cases, IGF-2 may even yield physical changes like those of acromegaly [3]. Here, we describe a case of ectopic IGF-2 production from a malignant hemangiopericytoma and discuss the management of resultant hypoglycemia.

## Case presentation

A 49-year-old Caucasian woman was diagnosed with an anaplastic meningioma 13 years before presentation. It was initially managed with surgery and adjuvant radiotherapy. Pathology was consistent with hemangiopericytoma. Six years before presentation, she was found to have multiple spinal lesions and underwent laminectomy with pathology definitively demonstrating metastasis of the original tumor. Subsequent imaging over the next 2 years revealed diffusely metastatic disease involving the axial skeleton, and multiple abdominal sites including the liver. In this interval, and the years leading up to presentation, she was treated with several systemic chemotherapy agents including dasatanib, avastin/ temozolomide, pazopanib, ponatinib and eribulin. In addition, she underwent ten rounds of palliative radiotherapy directed at metastases in the skeleton, liver and extra-hepatic abdominal sites. Disease continued to progress despite therapy; however, the patient maintained a high performance status, with stable weight, energy level, and basic laboratory testing. Indeed, she continued to work at a demanding professional job until 2 weeks before admission.

In the 2 months before presentation, the patient was noted to have hypoglycemia on basic laboratory evaluation, including a glucose value of 48 mg/dL (2.7 mmol/L) 3 weeks prior to admission. Three days before presentation, she was found diaphoretic, and unresponsive. She was taken to an outside emergency department, and found to be hypoglycemic. She was discharged with instructions to take juice for symptomatic hypoglycemia. On the day of presentation, she had a witnessed seizure, and was taken to our emergency department by ambulance. On arrival, she was found to have a blood glucose level of 18 mg/dL (1.0 mmol/L). Biochemical analysis was significant for low levels of insulin (<2μU/mL), C-peptide (0.1 ng/mL), and beta-hydroxybutyrate (<0.2 mmol/L). Random cortisol was 21 μg/dL, while, thyroid stimulating hormone (TSH) and free thyroxine (T4) were within normal limits. Insulin antibodies, and sulfonylureas were undetectable. IGF-1 level was 56 ng/mL (reference range 56–194 ng/mL), and IGFBP-3 was 1.2 μg/mL (reference range 3.3–6.7 μg/mL), while IGF-2 was markedly elevated at 1320 ng/mL (reference range 288–736 ng/mL). IGF-2: IGF-1 ratio was 24:1. The patient was treated acutely with 75 g dextrose (given as 50% solution (D50)), and euglycemia was maintained on 10% dextrose (D10) infusion.

The patient was then started on dexamethasone 6 mg twice daily, and took frequent small meals during the day. In addition to steroids, she started a diet rich in complex carbohydrates including uncooked corn starch (given at bedtime). She continued to have blood glucose levels as low as 60 mg/dL (3.33 mmol/L) on overnight fingerstick checks (performed every 2 h), prompting our decision to institute continuous glucose monitoring with a Dexcom G4 Professional CGMS. The system was set to alarm at blood glucose value <70 mg/dL (3.9 mmol/L). In addition, given patient’s ultimate preference for a steroid-sparing anti-hypoglycemia regimen, we reduced dexamethasone to 2 mg twice daily after starting nighttime subcutaneous glucagon (Lilly brand) infusion via Medtronic insulin pump—an approach initially described by Houlbert et al. [6]. We started infusion at 0.3 mg/ h based on average glucagon infusion rate employed in dual chamber insulin pump studies [7]. The patient was discharged on this regimen, and CGMS readings were reviewed at outpatient follow-up appointments.

**Fig. 1:**
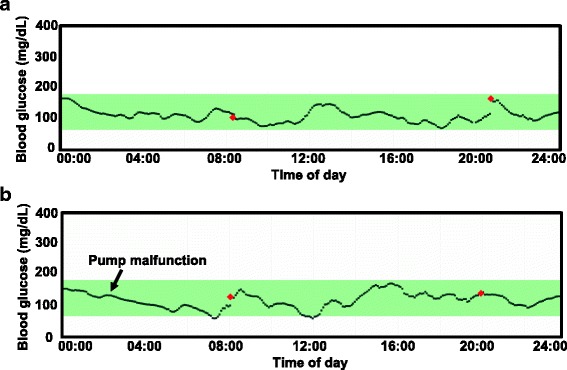
CGMS tracings from individual days, demonstrating (**a**) maintenance of euglycemia throughout the day. In the setting of pump dysfunction (**b**), overnight glucagon delivery ceased, precipitating hypoglycemia

**Fig. 2:**
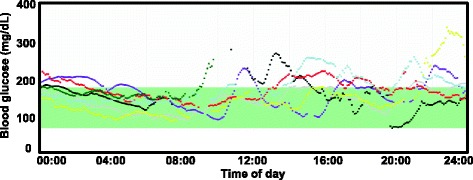
CGMS tracings taken over 1 week after initiation of everolimus. Each color represents an individual day

Review of the data demonstrated maintenance of euglycemia overnight (Fig. 1a). Dependence on glucagon infusion was demonstrated by blood glucose decline during a reported instance of pump technical malfunction (Fig. 1b). In response to persistent low-normal range blood glucose values, glucagon infusion rate was increased to 0.6 mg/ h. One month after initial presentation, the patient was started on everolimus 5 mg daily, resulting in marked improvement in hypoglycemia (Fig. 2). This enabled taper of dexamethasone dose to 0.5 mg twice daily, without further hypoglycemic episodes (while maintained on aforementioned overnight glucagon infusion). Recurrent herpes simplex (HSV) rashes emerged 1–2 weeks after starting everolimus; prompting its discontinuation after a total of 1 month therapy.

**Fig. 3:**
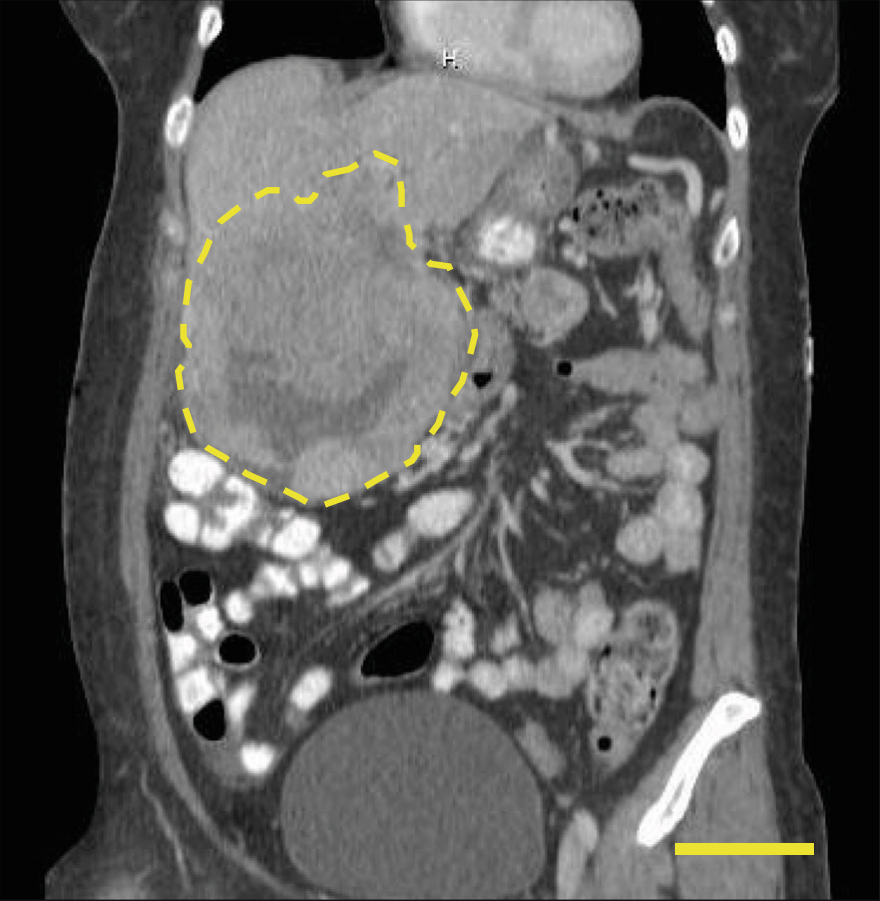
CT scan of causative upper right quadrant mass (indicated with dashed line). Scale = 5 cm

Abdominal CT scan demonstrated a right upper quadrant mass adjacent to, but not infiltrating the liver, that had increased in size to 14 × 11 cm from 11 × 9 cm on CT two months prior (Fig. 3). Given that this mass had not previously been subjected to radiotherapy, it was considered the putative source of IGF-2 production. The patient received 20 gray (Gy) radiation over five sessions. Following this definitive therapy, hypoglycemia resolved and IGF-2 level, measured two weeks after final radiation treatment, declined to 380 ng/mL.

## Discussion and conclusions

IGF-2omas are rare tumors, with incidence thought considerably lower than the 4 per million patient years cited for insulinoma [3]. The heterogeneous array of IGF-2 producing tumors contains multiple sarcomas, including a recently-described malignant renal solitary fibrous tumor [8], and hemangiopericytoma. Initial descriptions of hemangiopericytoma-associated hypoglycemia date to the 1950s [9], and a recent literature review found these tumors accounted for 19 of 288 (7%) IGF-2omas reported between 1988 and 2013 [10].

Most IGF-2omas predominantly synthesize a variety of incompletely processed IGF-2 precursors, collectively termed “big IGF-2” [1, 4]. Big IGF-2, while not detectable on most commercial assays, can be bioactive [11] and often underlies the pathogenesis of hypoglycemia [5, 12]. Indeed, many IGF-2omas, including hemangiopericytomas, present with normal or even low mature IGF-2 levels [13, 14]. Nonetheless, in instances like the present case, measurably high mature IGF-2 levels [8, 15] may aid in making the diagnosis and monitoring response to therapy.

Hypoglycemia is the immediate life-threatening complication of elevated IGF-2. The underlying mechanism is multifactorial, but thought to depend largely on binding of IGF-2 (or big IGF-2) to the insulin receptor with resultant stimulation of glucose uptake into skeletal muscle and suppression of glucagon secretion from the α-cell [3, 4, 16]. Notably, elevated IGF-2 activity suppresses both insulin and GH. The latter effect, mediated by IGF-2 binding to type I IGF receptors on pituitary somatotrophs, causes reduced levels of IGF-1 and IGF-BP3 [3, 4] and impairs the GH-mediated counter regulatory response [16]. Given the impact of IGF-2 on the somatotroph axis, an elevated IGF-2: IGF-1 > 10 has been found clinically suggestive of IGF-2-mediated hypoglycemia [3]. While it generally occurs in the fasting state, rare instances of post-prandial IGF-2-induced hypoglycemia have been anecdotally described [3].

Hypoglycemia demands urgent attention while definitive therapy of the primary tumor is planned and pursued. After initial management with glucose infusion, systemic glucocorticoids are the current mainstay of therapy [3]. Recombinant growth hormone has been used with some success [17], though presents the theoretical risk of enhancing tumor growth [3], and may ultimately cause increased insulin and IGF-1 levels [18]. Alternative steroid-sparing therapies such as mTor inhibitors, including everolimus, have demonstrated good clinical efficacy in insulinoma-mediated hypoglycemia [19], however, have not been extensively studied in the setting of IGF-2oma. While one report demonstrated hypoglycemia caused by an IGF-2-secreting adrenocortical carcinoma to be refractory to everolimus treatment [20], in the current case, this agent effectively mitigated hypoglycemia in the short term. Undesirable immunosuppressive side effects (recurrent HSV infection), however, forced its discontinuation after 1 month. Intravenous glucagon infusion has proven a valid complement for addressing fasting hypoglycemia mediated by IGF-2-producing tumors [10]. Nonetheless, it as been associated in some cases with side effects analogous to glucagonoma syndrome, including venous thromboembolism, necrolytic migratory erythema, and angular cheilitis [21]. Subcutaneous glucagon infusion, initially described by Houbert et al. [6], allows easier application of this therapy in the outpatient setting, and, in our hands caused no untoward effects. Notably, patients receiving glucagon must consume adequate carbohydrate load while awake in order to build sufficient glycogen reserve for mobilization by glucagon during sleep [6]. Multiple case reports and trials demonstrate that diazoxide and octreotide have no role in hypoglycemia caused by IGF-2omas [10].

In the case at hand, along with glucocorticoids, we instructed the patient to take a diet rich in complex carbohydrates, including a bedtime snack containing uncooked corn starch. Effecting a protracted glycemic peak versus other carbohydrate preparations, cornstarch has been shown to delay the nadir of overnight hypoglycemia in type 1 diabetic patients treated with basal-bolus insulin regimen or insulin pump [22], and mitigate hypoglycemic events in type 1 diabetic patients receiving bedtime NPH insulin [23]. An adjunctive therapy only in the diabetic population, uncooked cornstarch is unpalatable to many patients; and therefore can be given in the form of commercially-available supplement bars.

The use of glucagon, dispensed via insulin pump, is non-formulary, but was effective in this case. It is critical to note that glucagon formulations used in dual chamber insulin pump trials [7] are not yet commercially available. Though specific manufacturer guidelines do not exist for this application, we observed Lilly brand glucagon is preferable for pump administration but must be loaded into the reservoir at a concentration ≤ 0.5 mg/mL to prevent occlusion of the tubing—demonstrated by inability to pass solution during a bolus with the pump disconnected. In our hands, Novo Nordisk brand glucagon caused occlusion at or below this concentration.

Glucose monitoring with CGMS was critical in the current case to alert the patient of overnight hypoglycemia and to titrate doses of anti-hypoglycemic agents. Increasingly employed in the American type 1 diabetic population, CGMS has recently been used in the management of IGF-2-mediated hypoglycemia [24]. It is important to note that acetaminophen interferes with the CGMS glucose sensor and is therefore contraindicated for patients on these systems.

Management options for IGF-2oma-mediated hypoglycemia are summarized in (Table 1).

**Table 1:** Management Options for IGF-2oma-mediated Hypoglycemia

Therapy	Comment
Non-pharmacologic	
Glucose pushes/ infusion	Initial temporizing management in hospital setting
Dietary modifications	May be sufficient alone for mild hypoglycemia. Corn starch given at bedtime delays onset of overnight hypoglycemia and may be employed as adjunctive therapy in cases of more severe hypoglycemia. Tube feeds may have utility in the hospital setting/ while preparing for definitive management.
- Frequent small meals	
- Complex carbohydrates (corn starch)	
- Enteral nutrition	
Continuous glucose monitoring system	Useful for alerting patient to hypoglycemic events (particularly overnight) and for titrating efficacy of therapy. Tylenol interferes with glucose sensor; therefore, should be excluded form pain management regimen
Pharmacologic	
Glucocorticoids	Current mainstay of therapy. Inexpensive and effective. Must consider many short and long-term side effects.
Recombinant growth hormone	Possible adjunct to glucocorticoids; occasional efficacy as monotherapy. Theoretical risk for increased tumor growth.
mTOR inhibitors	Good efficacy in insulinoma. Not extensively investigated in IGF-2oma, however, successful in this case. Must consider immunosuppressive side effects.
Glucagon infusion	Effective in preventing overnight hypoglycemia. Must ensure adequate carbohydrate intake to replete hepatic glycogen stores during waking hours. Commercially available glucagon preparations not designed for subcutaneous infusion via pump; therefore, concentration in reservoir must be sufficiently low to prevent line occlusion. Best utilized in monitored setting—inpatient or with home CGMS. Notably, side effects including venous thromboembolism, necrolytic migratory erythema, and angular cheilitis, have been reported in patients receiving intravenous glucagon infusion.
Octreotide and diazoxide	No role in management of hypoglycemia caused by IGF-2oma

To our knowledge, this is the first instance in which a patient with an IGF-2 producing neoplasm has been managed with combination of subcutaneous glucagon infusion and CGMS; and also represents a novel use of everolimus (though the latter was discontinued due to side effects). In the case at hand, this strategy enabled complete avoidance of further major hypoglycemic events and streamlined titration of other anti-hypoglycemic agents. We feel that, while not yet standard of care for management of this rare oncologic complication, combined use of subcutaneous glucagon and CGMS has considerable value. Moreover, everolimus warrants further consideration as therapy for IGF-2oma-induced hypoglycemia.

## Abbreviations

CGMS: Continuous glucose monitoring system
CT: Computed tomography
D10: 10% dextrose solution
D50: 50% dextrose solution
FT4: Free thyroxine
Gy: Gray
HSV: Herpes simplex virus
IGF-1: Insulin-like growth factor-1
IGF-2: Insulin-like growth factor-2
IGFBP-3: Insulin-like growth factor binding protein-3
TSH: Thyroid stimulating hormone
